# Case Report: Ocular Manifestations and Treatments of Ciliary Body Involvement by Lymphoma

**DOI:** 10.3389/fonc.2021.718759

**Published:** 2021-09-23

**Authors:** Yu Di, Junjie Ye, Ruoan Han, Mengda Li, Bilei Zhang

**Affiliations:** ^1^ Department of Ophthalmology, Peking Union Medical College Hospital, Chinese Academy of Medical Sciences, Beijing, China; ^2^ Department of Ophthalmology, Beijing Tsinghua Changgung Hospital, School of Clinical Medicine, Tsinghua University, Beijing, China

**Keywords:** ciliary body, lymphoma, uveitis, diagnostic vitrectomy, treatment

## Abstract

**Purpose:**

To describe the ocular clinical features, histopathological findings, and treatment outcomes of lymphomas involving the ciliary body.

**Methods:**

We demonstrate three cases of ciliary body involvement by lymphoma from 2013 to 2019 in Peking Union Medical College Hospital (PUMCH). All patients underwent examinations including best corrected visual acuity (BCVA), slit-lamp microscopy, indirect ophthalmoscope, ultrasound biomicroscopy (UBM), and diagnostic vitrectomy. In addition, cytopathology, immunohistochemistry, gene rearrangement, cytometric immunophenotypic, or in-situ hybridization were used for determining the pathological type of lymphoma.

**Results:**

The patients were a 25-year-old man, a 52-year-old woman, and a 54-year-old man. Two patients had unilateral involvement, and one patient had bilateral involvement. All patients presented with anterior uveitis and elevated intraocular pressure. Ciliary body masses or infiltration were found in 3 patients. Two patients had diffuse large B-cell lymphoma and one patient had natural killer/T-cell lymphoma. All patients received 0.4 mg methotrexate intravitreal injections, and the ciliary body lesions regressed completely.

**Conclusion:**

Lymphomatous involvement of the ciliary body usually presents as an atypical anterior chamber reaction. Vitreous biopsy should be considered in these patients for diagnosis. Methotrexate intravitreal injection combine with chemotherapy or radiotherapy, might extend the survival time and preserve visual acuity for patients with ciliary body involvement by lymphoma.

## Introduction

Intraocular lymphoma is anatomically classified into vitreoretinal lymphoma, which is the most common type and is often associated with the central nervous system, and uveal lymphoma, which is further subdivided into choroidal, iridal, and ciliary types (1). The majority of uveal lymphomas are confined to the choroid, while reports of ciliary body involvement have rarely been documented. This rare condition is further classified as primary or secondary lymphoma, depending on whether the disease is confined to the ciliary body or whether there is concomitant extensive systemic lymphoma (2–8). We herein report the clinical features, histopathological findings, systemic associations and treatment outcomes of three cases of ciliary body involvement by lymphoma with different primary lesions.

## Case 1

A 25-year-old man was referred to our hospital in March 2013 with anterior chamber inflammation, vitreous opacity, and increased intraocular pressure (IOP) in his left eye. Over the previous five months, he had been admitted to a local hospital with cataract and vitreous hemorrhage in the left eye and underwent combined phacoemulsification, intraocular lens implantation and vitrectomy. However, there was no significant improvement in his left eye. His medical history included a diagnosis of primary central nervous system lymphoma (PCNSL) of diffuse large B-cell lymphoma (DLBCL) based on immunohistochemistry of a brain biopsy in March 2010. He noted that he had completed seven courses of chemotherapy [methotrexate (MTX), vincristine, ifosfamide, and dexamethasone] followed by one course of brain radiotherapy a period of 19 months. The volume of the brain lesions decreased significantly.

**Figure 1:**
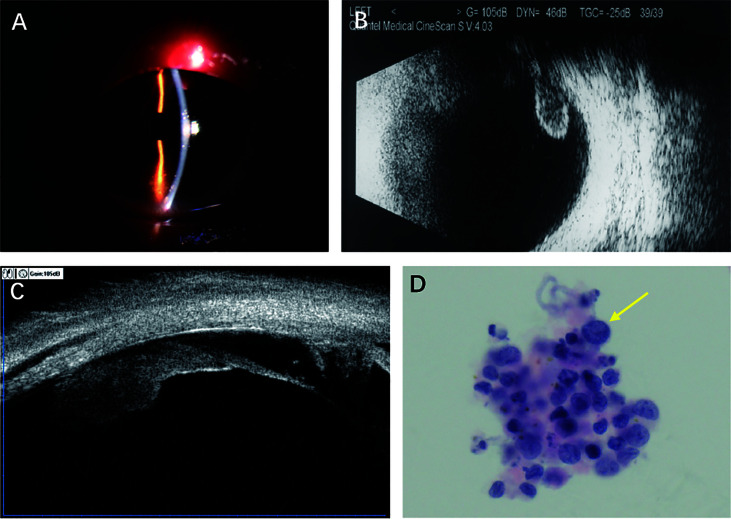
Findings from a 25-year-old man presenting with anterior chamber inflammation, vitreous opacity, and increased intraocular pressure in the left eye. **(A)** Slitlamp photograph showing anterior chamber reaction (keratic precipitates 1+, flare 1+, cell 1+). **(B)** B-Scan revealing vitreous opacity and a plurality of white lumps on the surface of inferior retina. **(C)** Ultrasonographic biomicroscopy showing local masses with acoustic solidity but low internal reflectivity. **(D)** Liquid-based cytology test (hematoxylin-erosin; original manification, x 40) showing large atypical lymphoid cells.

Our ophthalmological examination revealed an elevated IOP (34.1 mmHg, 1 mmHg=0.133 kPa), anterior chamber reaction (keratic precipitates 1+, flare 1+, cell 1+) (**Figure 1A**), vitreous opacity and a plurality of white lumps on the surface of the inferior retina in the left eye. While there were no significant lesions in the fundus. The best corrected visual acuity (BCVA) was 20/16 and 20/200 in his right and left eyes, respectively. B-scan revealed vitreous opacity and epiretinal deposits, and ultrasound biomicroscopy (UBM) found a ciliary body mass with almost 360° involvement in the left eye (**Figures 1B, C**).

Diagnostic vitrectomy was performed in the left eye on April 15th, 2013. Liquid-based cytology revealed large atypical lymphoid cells that were CD20+ on flow cytometry (**Figure 1D** and **Table 1**). Brain magnetic resonance imaging, cerebrospinal fluid and bone marrow were insignificant. The patient received ocular intensity-modulated radiation therapy (IMRT, 45 Gy) and a series of 0.4 mg MTX intravitreal injections (weekly for the first month, every 2 weeks for the second month, and monthly for 10 months). After one-month treatment with 4 intravitreal injections of MTX, his condition was assessed again. His BCVA improved to 20/40, the IOP was reduced to 16 mmHg, and the anterior inflammation disappeared in the left eye. UBM reexamination showed complete regression of the ciliary body lesions. Then, the patient completed remained 12 intravitreal injections of MTX, and there was no recurrence of the intraocular lymphoma during the one-year follow-up period.

**Table 1:** Diagnostic methods of ciliary body lymphoma.

Case	Diagnostic Methods				Intraocular lymphoma	Systematic lymphoma	
	Cytopathology	Immunohistochemistry	Gene rearrangement	Cytometric immunophenotypic analysis	Pathologic type	Location	Pathologic type
1	atypical lymphoid cells	–	–	CD38+, CD19+, CD20+, HLA-DR+	DLBCL	CNS	DLBCL
2	small malignant cells	CD2(+), Ki-67(index 85%), CD3(+), CD4(+), CD56(+), granzymeB(+)	TCRG+	–	NK/T-cell lymphoma	nose	NK/T-cell lymphoma
3	atypical lymphoid cells	–	IgH+,IgK+	–	DLBCL	paranal sinus	DLBCL

DLBCL, diffuse large B cell lymphoma; CNS, central nervous lymphoma.

## Case 2

A 52-year-old woman presented to our hospital with a 1-month history of visual deterioration in the right eye on July 20th, 2020. She was diagnosed as anterior uveitis in a local hospital and treated with prednisolone eye drops. However, the ocular symptoms were not relieved, so she was referred to a higher-tier hospital. Her medical history included a diagnosis of natural killer/T-cell lymphoma (NKTL) nasal type [CD20 (–), CD3(+), CD56(+), TIA-1(+)] based on immunohistochemistry of a nasal biopsy in April 2020, and she was treated with nasal radiotherapy from May 2020 to June 2020.

**Figure 2:**
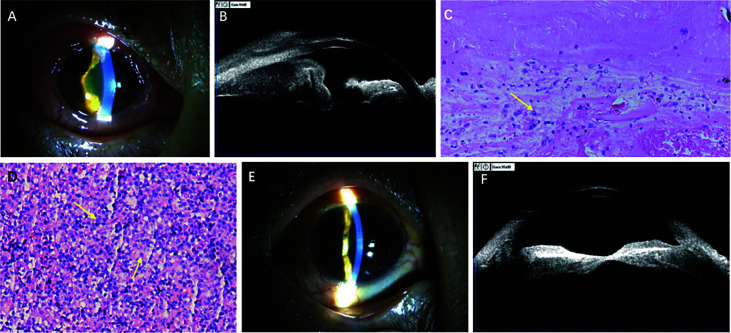
Findings from a 52-year-old woman presenting with visual deterioration in the right eye. **(A)** Slitlamp photograph showing conjunctival congestion and edema, anterior chamber reaction (keratic precipitates +, flare 2+, cell 1+), posterior synechia of the inferior part of the iris, and superior nasal part of the peripheral anterior chamber obliterated. **(B)** Ultrasonographic biomicroscopy revealing 360 ° infiltration of conjunctiva, iris, and ciliary body with low and medium internal reflectivity. **(C)** Photomicrograph of vitreous biopsy (hematoxylin-erosin; original manification, x 200) showing small malignant cells. **(D)** Photomicrograph of conjunctival biopsy (hematoxylin-erosin; original manification, x 200) showing small malignant cells. **(E)** Slitlamp photograph demonstrating conjunctival congestion and edema, anterior chamber reaction relieved significantly after treatment. **(F)** Ultrasonographic biomicroscopy showing lesions of ciliary body basically disappeared after treatment.

On admission, her BCVA was finger count (FC) in the right eye and 20/32 in the left eye, and the IOP was 31 mmHg and 18 mmHg in the right and left eyes, respectively. Slit-lamp examination of the right eye revealed anterior chamber reaction (keratic precipitates+, flare 2+, cell 1+), posterior synechia of the inferior part of the iris, and obliteration of the superior nasal part of the peripheral anterior chamber (**Figure 2A**). Dilation examination revealed an invisible fundus. B-scan of the right eye revealed vitreous opacity and retinal detachment. UBM of the right eye revealed 360° infiltration of the conjunctiva, iris, and ciliary body with low and medium internal reflectivity, more prominent on the superior nasal side (**Figure 2B**).

Diagnostic vitrectomy and biopsy of the bulbar conjunctiva were performed in the right eye on July 27, 2020. Pathology revealed small malignant cells in the vitreous humor and bulbar conjunctiva (**Figures 2C, D**), which were then confined to be NKTL in origin by immunohistochemistry [CD2(+), Ki-67(index 85%), CD3(+), CD4(+), CD56(+), granzymeB(+)], gene rearrangement studies [TCRG(+)], and in-situ hybridization [EBER(+)] (**Table 1**). Given her past medical history, intraocular NKTL was diagnosed. Brain magnetic resonance imaging was insignificant. Then, the patient received a series of 0.4 mg MTX intravitreal injections (the specific therapeutic regimen was the same as for **Case 1**). Additionally, the patient was transferred to the hematology department of our hospital for chemotherapy [steroids (dexamethasone), MTX, ifosfamide, L-asparaginase, etoposide]. After two months with 3 courses of chemotherapy and 6 intravitreal injections of MTX, the symptom of blurred vision and pain were relieved, slit-lamp examination of the left eye revealed the anterior inflammation disappeared in the left eye. UBM reexamination showed complete regression of the ciliary body lesions (**Figures 2E, F**). Position emission tomography-computer tomography (PET-CT) of the patient also revealed low metabolic foci in the right eye, which indicated that the lesion of the right eye had basically disappeared. The patient completed 14 intravitreal injections of MTX during the 9-month follow-up period. Unfortunately, the patient died of nasal NKTL recurrence in April 2021.

## Case 3

A 54-year-old man presented to our hospital with redness, pain and blurred vision in both eyes in July 2019. In the previous three months, he was admitted to a local hospital with anterior uveitis and secondary glaucoma in both eyes and was given prednisolone and IOP-lowering eye drops. However, both eyes further deteriorated, and the patient presented with hypopyon and increased IOP. His medical history included a diagnosis of systemic DLBCL based on immunohistochemical analysis of an axillary lymph-node biopsy in December 2018. He has received six courses of chemotherapy (standard rituximab, cyclophosphamide, doxorubicin, vincristine, and prednisone), and his general condition is now stable.

**Figure 3:**
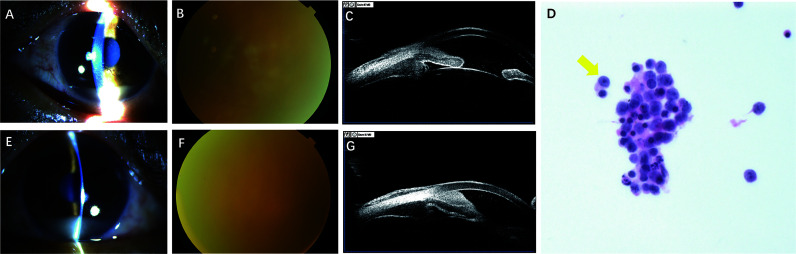
Findings from a 54-year-old man presenting with redness, pain and blurred vision in both eyes. **(A, E)** Slitlamp photograph showing severe anterior chamber reaction (keratic precipitates 3+, flare 1+, cell 4+), and a pseudohypopyon in the right and left eye, respectively. **(B, F)** Dilation examination revealing an invisible fundus in the right and left eye. **(C, G)** Ultrasonographic biomicroscopy revealing revealed 360° thickening of the iris and ciliary body, plus peripheral angle closure in the right and left eye, respectively. **(D)** Liquid-based cytology test of aqueous and vitreous humor (hematoxylin-erosin; original manification, x 40) showing atypical lymphoid cells.

His BCVA was 20/100 in the right eye and 20/200 in the left eye, and his IOP was 42 mmHg and 45 mmHg in the right and left eyes, respectively. Biomicroscopic examination of both eyes showed severe anterior chamber reaction (keratic precipitates 3+, flare 1+, cell 4+) and a pseudohypopyon in the inferior angle (**Figures 3A, E**). The bilateral peripheral anterior chamber was obliterated, and the iris appeared thickened with nodular areas of infiltration. Dilation examination revealed an invisible fundus. B-scan demonstrated clear vitreous and an absence of fundus anomalies (**Figures 3B, F**). UBM revealed 360° thickening of the iris and ciliary body, plus peripheral angle closure (**Figures 3C, G**). Paracentesis of the anterior chamber and diagnostic vitrectomy were performed in the left eye on July 22, 2019. Liquid-based cytology revealed atypical lymphoid cells (**Figure 3D**), which were IgK and IgH on gene rearrangement analysis (**Table 1**). Brain magnetic resonance imaging was unremarkable. The patient was given a series of 0.4 mg MTX (the specific therapeutic regimen was the same as for Case 1). Following a single MTX injection, the patient felt the symptom of ocular pain was relieved, the BCVA improved to 20/63 and 20/100 in the right and left eyes, respectively, the IOP decreased to 13 mmHg in the right eye and 19 mmHg in the left eye, and the slit-lamp examination revealed the pseudohypopyon had disappeared. After that, the patient received 6 intravitreal injections of MTX and 3 courses of chemotherapy. However, the patient lost to follow-up after 3 months of treatment.

## Discussion

Most symptoms (blurred vision, red eye, eye pain) and clinical findings (ciliary body injection, anterior chamber reaction, posterior synechiae) associated with ciliary body lymphoma overlap with those of anterior uveitis (3). Ahmed et al. (9) found that an anterior reaction and pseudohypopyon may be present, especially in patients with secondary ciliary body lymphoma. However, Mashayekhi et al. (3) proposed that pseudohypopyon is not clinically distinguishable from the true hypopyon found in anterior uveitis. Ciliary body lymphoid infiltration is a useful clinical feature for differentiating ciliary body lymphoma from anterior uveitis. UBM of the anterior segment is a helpful technique for assessing the deeper ocular structures such as the ciliary body (10). For anterior uveitis, ciliary body inflammatory exudates and fibrosis were common manifestations in the UBM examination (11). However, for ciliary body involvement by lymphoma, UBM showed local masses or diffuse infiltration of the ciliary body with acoustic solidity but low internal reflectivity (12). Thus, we can differentiate ciliary body lymphoma from anterior uveitis through the ciliary body manifestations with UBM. However, pathological results are important basis for diagnosis.

The identification of lymphoma cells is considered the gold standard for the diagnosis of lymphomatous involvement of ciliary (5). Pei et al. (6) proposed that the presence of tumor cells in the aqueous humor makes it possible to diagnose ciliary body involvement by lymphoma. However, aqueous samples are frequently nonconfirmatory due to the scant cellularity of the sample (13, 14). Diagnostic vitrectomy has several advantages, including improved vision following clearance of vitreous debris and maximization of the sample size (15). All of our patients underwent diagnostic vitrectomy to identify malignant lymphocytic cells, and the pathological types were confirmed by liquid-based cytology or gene rearrangement.

For the treatment of ciliary body involvement by lymphoma, the standard and optimal therapy has not been defined. Intravitreal injections of MTX have been found to be effective in inducing clinical remission of intraocular lymphoma, and Frenkel et al. (16) proposed that it can be used as a first-line treatment for the disease. Additionally, chemotherapy and radiotherapy should be used for the ocular lymphoma treatment and are considered an effective treatment to control primary lesions. All of our patients received a series of 0.4 mg MTX intravitreal injections. Case 2 and Case 3 referred to the hematology department for chemotherapy after ocular treatment, but Case 1 only combined with ocular intensity-modulated radiation therapy (IMRT), because the patient’s brain magnetic resonance imaging revealed no significant lesions after seven courses of chemotherapy. And there was no recurrence of the intraocular lymphoma during the one-year follow-up period. In all treated eyes, clinical remission of the ciliary body lymphoma was demonstrated during the follow-up by improvement of BCVA, decrease of IOP, disappearance of anterior segment inflammation, and resolution of ciliary body infiltration.

Zhou et al. (17) proposed a modified “intensive-consolidation-maintenance” regimen for treatment of primary vitreoretinal lymphoma. If patients achieved remission before 7 injections, they would stop the injections; if patients did not achieve remission after full course of 7 injections, they would start another course of 7 injections until achieving the clinical remission. However, there are no precise criteria to terminate treatment of the ciliary body involvement by lymphoma. In our study, all treated eyes should complete 16 intravitreal injections through 1-year treatment. One-month follow-up after the last injection and patient could see a doctor immediately in case of discomfort. Case 1 patient completed full course of 16 injections, and there was no recurrence of the intraocular lymphoma during the follow-up. Case 2 received 14 intravitreal injections due to the recurrence of nasal NKTL. Case 3 only completed 7 intravitreal injections because of the loss of follow-up after 3-month treatment. Reversible keratopathy was the most prevalent side-effect of intravitreal MTX (18). Smith et al. (19) reported other complications, such as neovascularization glaucoma, optic atrophy and maculopathy. Maculopathy, associated with significantly irreversible vision loss, may be related to intravitreal injection of MTX. In our study, treatment with intravitreal injection of MTX not resulted in any ocular or systematic complications.

## Limitations

Due to the rarity of ciliary body lymphoma, our case series only included 3 patients for clarifying ocular manifestations and treatments of this disease. A series involving a larger number of patients from multiple centers may help further elucidate the details in these findings.

## Conclusions

In conclusion, we report the clinical and histopathological findings of 3 cases of lymphoma affecting the ciliary body. Clinicians should be aware that ciliary body lymphoma usually presents as an atypical anterior chamber reaction, which can be misdiagnosed as anterior uveitis. Ciliary body lymphoid infiltration is a useful clinical feature for differentiating ciliary body lymphoma from anterior uveitis. UBM is useful for the evaluation of ciliary body lymphoma, and vitreous biopsy should be considered in these patients for diagnosis. Intravitreal injection of MTX is a first-line treatment for intraocular lymphoma that, in combination with chemotherapy or radiotherapy, might extend the survival time and preserve visual acuity for patients with ciliary body lymphoma.

## Data Availability Statement

The raw data supporting the conclusions of this article will be made available by the authors, without undue reservation.

## Ethics Statement

The studies involving human participants were reviewed and approved by the Institutional Review Board/Ethics Committee of Peking Union Medical College Hospital. The patients/participants provided their written informed consent to participate in this study. Written informed consent was obtained from the individual(s) for the publication of any potentially identifiable images or data included in this article.

## Author Contributions

All authors have approved this submission to Frontiers in Oncology. YD carried out the entire procedure including the collection of medical records, drafting the manuscript and manuscript revision. JY conceived of the study, coordinated and participated in the entire process of drafting and revised the manuscript. RH, ML, and BZ were involved directly in the care of patients. All authors contributed to the article and approved the submitted version.

## Conflict of Interest

The authors declare that the research was conducted in the absence of any commercial or financial relationships that could be construed as a potential conflict of interest.

## Publisher’s Note

All claims expressed in this article are solely those of the authors and do not necessarily represent those of their affiliated organizations, or those of the publisher, the editors and the reviewers. Any product that may be evaluated in this article, or claim that may be made by its manufacturer, is not guaranteed or endorsed by the publisher.
